# FOXC1-induced LINC01123 acts as a mediator in triple negative breast cancer

**DOI:** 10.1186/s12935-020-01258-z

**Published:** 2020-05-29

**Authors:** Purong Zhang, Qimin Long, Shiyan Zeng, Min Wen, Qing Lu

**Affiliations:** 1 Department of Breast Surgery, West China Hospital/West China School of Medicine, Sichuan University, No. 37, Guoxue Lane, Wuhou District, Chengdu, 610041 People’s Republic of China; 2 Sichuan Cancer Hospital & Institute, Sichuan Cancer Center, School of Medicine, University of Electronic Science and Technology of China, Chengdu, 610041 People’s Republic of China

**Keywords:** FOXC1, LINC01123, miR-663a, CMIP, TNBC

## Abstract

**Background:**

MicroRNAs (miRNAs) representing a subclass of non-coding RNAs are dynamically expressed and participate in multiple pathological responses, whereas, the expression pattern or function of miRNAs has not been fully addressed in triple-negative breast cancer (TNBC). Currently we concentrate on dissecting the probable role of microRNA-663a (miR-663a) in TNBC cellular processes.

**Methods:**

qRT-PCR detected the expression of miR-663a in TNBC cells. Besides, we monitored the effects of miR-663a on TNBC proliferation and apoptosis. On the basis of bioinformatics assistance and mechanical validation, we identified the miRNA-sponging role of LINC01123 and downstream target of miR-663a in TNBC was assessed and verified. The transcription activation of was explored via ChIP and luciferase reporter assays.

**Results:**

In comparison to MCF-10A, we certified the downregulation of miR-663a in TNBC cell lines. Augmentation of miR-663a was anti-proliferation and pro-apoptosis in TNBC cell lines. LINC01123 protected CMIP against miR-663a suppression through acting as a sponge of miR-663a in TNBC. LINC01123 was transcriptionally induced by FOXC1. Rescue experiment proved that miR-663a suppression or CMIP (c-Maf inducing protein) enhancement could countervail LINC01123 depletion-mediated effects on TNBC cellular processes.

**Conclusion:**

LINC01123, activated by FOXC1, regulated TNBC growth through miR-663a/CMIP signaling, which unveiled a new functional pathway of FOXC1-induced LINC01123/miR-663a/CMIP in TNBC.

## Background

Triple-negative breast cancer (TNBC) is generally identified as a malignancy with high aggressive property. Continually, TNBC causes severe health concern among females [1]. 12 to 17% of breast tumor cases are identified as TNBC, which are featured by negative expression of human epidermal growth factor receptor-2 (HER-2) and hormone receptor (HR) [2]. Even TNBC only occupies a small percentage of all breast cancer cases, a severe challenge is posed in clinically treating patients with TNBC. Typically, younger populations are susceptible to be diagnosed with TNBC. Moreover, there is a great chance for patients to develop larger tumors, accompanied with the increased possibility of distant aggressiveness and death [3, 4]. Thus, urgent elucidation of the molecular mechanisms around TNBC progression are needed for the purpose of identifying available therapeutic molecules.

In the studies of tumor biology, long non-coding RNAs (lncRNAs) are emerged as molecular and clinical biomarkers in combating tumor exacerbations [5, 6]. According to its definition, it refers to transcripts whose length is above 200nt without protein-coding potential [7]. Initially, the generation of lncRNAs is viewed as useless during the process of transcription [8]. Recently, the broad regulation of lncRNAs has become more and more evident during a series of cellular biological functions [9]. To date, massive lncRNAs are corroborated to drive many essential cancerous phenotypes via their interactions with other cellular regulatory molecules [10]. For instance, lncRNA AFAP1-AS1 induces osteosarcoma tumorigenesis and stimulates epithelial-mesenchymal transition via modulating RhoC/ROCK1/p38MAPK/Twist1 pathway [11]. It was revealed that lncRNA-CF129 suppresses pancreatic cancer aggravation via the transcription repression of FOXC2 [12]. Accumulating evidence displayed that lncRNAs residing in nucleus could participate in modulating transcriptional activation, heterochromatin formation, X chromosome inactivation, and maintaining telomeres [13, 14]. On the other hand, lncRNAs pervasively locating within cytoplasm or shuttling between cytoplasm and nucleus also play their essential parts in mediating the translation or decay of messenger RNAs (mRNAs), and trafficking cytoplasmic protein [15, 16]. It is well-exemplified that cytoplasmic lncRNAs compete for the binding to miRNAs with mRNAs and therefore lessen miRNA-induced inhibition on targeted mRNAs, giving rise to mRNA increase [17]. A series of lncRNAs have been underscored. LncRNA SLC25A5-AS1 restores the expression and function of PTEN via acting as a miR-19a-3p in suppressing malignant phenotypes in gastric cancer [18]. LncRNA SNHG20 facilitates the downstream target ZEB2 and RUNX2 via sponging miR-154 in promoting non-small cell lung cancer progression [19]. Herein, the expression specificity of LINC01123 (long intergenic non-protein coding RNA 1123) has not been revealed in TNBC. Whether it functions in TNBC or not needs precise investigations.

In addition, the specific expression of lncRNAs is attributed to the transcriptional manipulation of diverse transcriptional factors. Prior studies validated that LINC00460 is a PRDX1-initiated lncRNA in head and neck squamous cell carcinoma [20]. C8orf76 directly binds to lncRNA DUSP5P1 promoter and induces lncRNA expression in gastric cancer [21]. However, the transcription of LINC01123 in TNBC remains obscure.

Therefore, our study focused on the biological function of LINC01123 in TNBC and its possible regulatory mechanism, which may enrich the basis for TNBC oncology.

## Materials and methods

### Cell culture

The cell line of normal mammary epithelial (MCF-10A) and cell lines of human breast cancer (MDA-MB-231, MDA-MB-468, MCF7, HCC1937) were received from the purchasing channel of American Type Culture Collection (Manassas, VA, USA). 10% fetal bovine serum (FBS, Invitrogen, Carlsbad CA, USA) was added into RPMI1640 medium (Invitrogen) for cultivating MDA-MB-468 and HCC1937 cells at 37 °C and in 5% CO_2_. DMEM medium (Invitrogen) was used to incubate MDA-MB-231 and MCF7 cells with 10% FBS under the temperature of 37 °C and 5% CO_2_. DMEM/F12 medium (Invitrogen) was used to cultivate MCF10A cells with 5% horse serum, 20 ng/ml EGF, 0.5 mg/ml Hydrocortisone, 100 ng/ml Cholera Toxin, 10 μg/ml Insulin under the temperature of 37 °C and 5% CO_2_. The use of Mycoplasma Plus PCR Primer Set (Agilent, Santa Clara, CA, USA) was used to detect the cells for mycoplasma contamination and the result was negative.

### Cell transfection

The amplified full-length LINC01123 or CMIP sequences were encompassed by the cutting sites of two *EcoRI.* Then the *EcoRI* linearized pIRSE2-EGFP vector was used to insert the fragments for constructing LINC01123 expression vector. Lipofectamine 2000 reagent (11668-019, Invitrogen) was utilized to conduct the transfection for 48 h using 10 mM vectors (10 nM) or 50 nM shRNAs with the consistence of 5 × 10^5^ cells. For stable transfection, the shRNAs were inserted into the lentivirus expression vector pCDH-CMV-MCS-EF1-Puro (System Bioscience, Palo Alto, CA, USA). 2 μg/ml of puromycin (Thermo Fisher, Waltham, MA, USA) was then added for screening out the stable cell lines. qRT-PCR was adopted to check the efficiency of transfection. The sequences of indicated shRNAs were presented as follows: sh/NC, 5′-CCGGGATTAGACCTGATAAGAATTATCTCGAGCTAATCTGGACTATTCTTAATATTTTTG-3′, sh/LINC01123#1, 5′-CCGGTCGGAAGCCCCTGTCGCGGTAGCTCGAGAGCCTTCGGGGACAGCGCCATCTTTTTG-3′, sh/LINC01123#2, 5′-CCGGGTGGAGCCAGCAGTCCCCGGCGCTCGAGCACCTCGGTCGTCAGGGGCCGCTTTTTG-3′; sh/NC, 5′-CCGGAAGTTATAGAACAAGAAGTAAACTCGAGTTCAATATCTTGTTCTTCATTTTTTTTG-3′, sh/CMIP#1, 5′-CCGGAGAGACAAACCAAATGGGCAGGCTCGAGTCTCTGTTTGGTTTACCCGTCCTTTTTG-3′, sh/CMIP#2, 5′-CCGGAGAGTCCTGGGTCGCCACCAGCCTCGAGTCTCAGGACCCAGCGGTGGTCGTTTTTG-3′; sh/NC, 5′-CCGGAAGTCAAGTTGATATAAATGTACTCGAGTTCAGTTCAACTATATTTACATTTTTTG-3′, sh/FOXC1#1, 5′-CCGGCGCCCTCTACAAGCTCAGTGTCCTCGAGGCGGGAGATGTTCGAGTCACAGTTTTTG-3′, sh/FOXC1#2, 5′-CCGGTGGGAGTTTCGGCTTGATTTAGCTCGAGACCCTCAAAGCCGAACTAAATCTTTTTG-3′.

### qRT-PCR

The use of TRIzol reagent (Invitrogen Life Technologies, Carlsbad, CA, USA) was to separate the total RNA from cultured cells. And then it was estimated by standard denaturing agarose gel electrophoresis and NanoDrop spectrophotometer ND-8000 (NanoDrop Technologies; Thermo Fisher). On the basis of the protocol of manufacturer, PrimeScript™ RT Master Mix (Takara Bio, Otsu, Japan) was used to compose cDNA via reverse transcription. And the total volume was 10 µL. Then, the reaction mix was deposited in a cryogenic environment of − 20 °C for future experiments after DEPC-Treated Water (Ambion^®^) was used to deliquate the reaction mix. SYBR^®^ Premix Ex Taq™ II (Takara Bio, Otsu, Japan) was used to conduct the quantitative real-time PCR in the PCR reaction mixture of 10 µl (containing 1 μl of cDNA). PCR conditions were comprised of pre-denaturation at 95 °C for 10 min, 40 cycles of denaturation at 95 °C for 15 s, annealing at 60 °C for 1 min and extension at 72 °C for 30 s. And the internal control was GAPDH. The use of ABI 7500 Real-Time PCR system (Applied Biosystems^®^) was to measure the transcript levels of all lncRNA. And then the fold change (FC) was identified by the 2^−ΔΔCt^ method. The specific PCR primers were listed as follows: miR-663a, Forward Primer, 5′-AGGCGGGGCGCCGCGGGACCGC-3′, Reverse Primer, 5′-CTCAACTGGTGTCGTGGA-3′; LINC01128, Forward Primer, 5′-GCCAGTGGAACATAAACCACC-3′, Reverse Primer, 5′-AGCCTGTCACAAACTGATTCT-3′; LINC01106, Forward Primer, 5′-GGAGCGCGTGCGATAATCT-3′, Reverse Primer, 5′-CTTGGAGTCGGTGAGAAGGC-3′; LINC01123, Forward Primer, 5′-GAACATGTGCTTGGTGTCGT-3′, Reverse Primer, 5′-AGCCACTTGCCTATGCGTG-3′; RUSC1-AS1, Forward Primer, 5′-TAACCCAATGACCCACCCAG-3′, Reverse Primer, 5′-AAAACGGAGCCCAGTTGGAA-3′; CMIP, Forward Primer, 5′-CAGCTCACGATTCCTGGGG-3′, Reverse Primer, 5′-CAGCGGCTTGGGTTACTCA-3′; LSP1, Forward Primer, 5′-GGAGCACCAGAAATGTCAGCA-3′, Reverse Primer, 5′-TCGGTCCTGTCGATGAGTTTG-3′; FOXC1, Forward Primer, 5′-GGCGAGCAGAGCTACTACC-3′, Reverse Primer, 5′-TGCGAGTACACGCTCATGG-3′; GAPDH, Forward Primer, 5′-GGAGCGAGATCCCTCCAAAAT-3′, Reverse Primer, 5′-GGCTGTTGTCATACTTCTCATGG-3′; U6, Forward Primer, 5′-CCAAATCTAGCTGCTGCGGT-3′, Reverse Primer, 5′-AGGTTTGTCGTTCCCGTCTC-3′.

### Cell counting kit-8 (CCK-8) assay

The cell samples at the logarithmic growth phase were collected from each group after 48 h of transfection, and then seeded into the 96-well plates with the cell density of 5 × 10^3^ cells in each well. 10 μl of CCK-8 solution (Dojindo Laboratories, Kumamoto, Japan) was added and cultured with cell samples for 2 h for assessing cell viability. At length, the optical density (OD) values at 450 nm were examined at indicated time points by use of spectrophotometer (Thermo Fisher Scientific, Waltham, MA, USA).

### Colony formation assay

After indicated transfection, 6-well cell petri dish was utilized to cultivate the cells at the logarithmic growth phase with the consistence of 2 × 10^4^ for 14 days at the temperature of 37 °C in 5% CO_2_. Then cells were rinsed by the use of PBS and fixed by the cold methanol for 30 min. After that, 0.005% crystal violet was utilized to stain the cells for 30 min. In the end, the quantity of colonies (with more than 50 cells) was manually calculated.

### EdU assay

The 12-well plate was adopted to seed the stably transfected cells. When the cells were fully adhered to each other, BeyoClick™ EdU Cell Proliferation Kit (Beyotime, Shanghai, China) was supplemented to every well after it was diluted, following the user guide. The cells were cultivated in an incubator which the temperature was 37 °C and the air contained 5% CO_2_. The process lasted for 2 h. Then PBS and 4% paraformaldehyde were utilized to rinse and fix the cells separately at RT for 20 min. After that, nuclei were stained by the utilization of DAPI. Finally, a fluorescence microscope (Olympus, Tokyo, Japan) was utilized to obverse the cells.

### Apoptosis assay

1 × 10^6^ cells were collected after transfection, and plated into the 6-well plates for cell apoptosis assay. The propidium iodide using an APC Annexin V Apoptosis Detection Kit (BioLegend, San Diego, CA, USA) and fluorescein isothiocyanate-Annexin V were utilized to stain the cells which were gathered before. After they were cultivated in the dark room for 15 min at the temperature of 37 °C, a flow cytometer (BD Biosciences, Franklin Lakes, NJ, USA) was adopted to take the analysis of the situation of cell apoptosis.

### TUNEL assays

The TUNEL assay was adopted to detect cell apoptosis via testing the fragmentation of apoptosis-induced DNA. 24-well flat-bottomed plates were utilized to cultivate the HCC1937 and MDA-MB-231 cells in 96-well plates separately and the concentration of each well was 1 × 10^5^ cells. Then 4% (v/v) paraformaldehyde was adopted to fix the cells for half an hour at 4 °C. On the basis of protocols of supplier, cells were then permeabilized with 0.1% Triton-X100. The in situ cell death detection kit (Roche, Basel, Switzerland) was used for the TUNEL staining and then DAPI was used to stain the nuclei for 10 min. The fluorescence microscope (Olympus) was utilized to detect the quantities of TUNEL-positive cells and ImagePro Plus software was adopted to estimate the rate of apoptosis cells.

### RIP assay

In accordance of suppliers’ instructions, Magna RIP™ RNA-Binding Protein Immunoprecipitation Kit (Millipore, Bedford, MA, USA) was used to conduct the RIP assay. Simply put, cells were subjected to trypsinization and then washed by the use of ice-cold PBS for two times. After that, RIP lysis buffer which was added with RNase inhibitor and protease inhibitor cocktail was utilized to resuspend the cells. Then the cells were taken to conduct the single freeze–thaw cycle for lysing gently. The 30 μl of magnetic beads were supplemented with antibody for cultivating in the RIP wash buffer at the temperature of 37 °C for half an hour with stirring. The cell lysates were supplemented into the beads for cultivating at 4 °C before the beads were rinsed by the utilization of RIP wash buffer for three times. And the process took one night. Then the proteinase K buffer was utilized to digest and conduct the resuspension to each immunoprecipitant for cultivating at 55 °C. This process lasted for half an hour. With the help of phenol, chloroform, and isoamyl, RNA was separated in line with the protocols of manufacturer and estimated by the adoption of qRT–PCR.

### Nuclear and cytoplasmic fractionation

On the basis of protocols of manufacturer, the utilization of the NE-PER™ Nuclear and Cytoplasmic Extraction Reagents was to conduct the nuclear/cytoplasmic isolation, as guided by supplier (Thermo Fisher Scientific). Cell samples were first lysed in cell fractionation buffer, and then centrifuged to collect cell cytoplasmic fraction. After that, cell samples were treated in cell disruption buffer, and then cell nucleus was obtained. Nuclear and cytoplasmic fractions were separated for the extraction of RNA. The utilization of GAPDH and U6 were served as the qRT-PCR markers separately for cytoplasmic and nuclear components.

### Western blot

On the basis of instructions of manufacturer, all proteins were abstracted by the utilization of RIPA solution containing RNase inhibitor and protease inhibitor cocktail. The concentration of protein was measured by the adoption of BCA method. Then 20 µg of protein was subjected to SDS-PAGE gel (10%) electrophoresis. Then skimmed milk (5%) was utilized to cultivate the PVDF membranes (Bio-Rad, Inc., Hercules, CA, USA) for blockading after the transfection was completed. Following, the membranes were subjected to overnight incubation with the relevant primary antibodies and GAPDH at the temperature of 4 °C. After that, the membranes were incubated with secondary antibodies for 3 h at room temperature. Then membranes were dropped with ECL luminous liquid (Sigma Aldrich; Merck KGaA, Darmstadt, Germany) for developing the signals. Finally, the densitometry was utilized to detect ECL chromogenic substrate.

### RNA pull-down assay

The pBluescript II SK (+) was utilized to clone the cDNA sequence of LINC01123. Biotin-labeled RNAs were subjected to the transcription and purification in vitro. Then the whole cell lysate which was included 1 mg proteins was utilizes to mix the biotinylated RNA and biotinylated RNA was then performed with Streptavidin agarose beads which were rinsed in TBS before. Then they were cultivated for 1 h at the temperature of 37 °C. TBS was used to rinse the beads for 5 min and SDS buffer was used to boil the beads. The RNA–protein mixture was eluted and digested for 2 h. Finally, western blot was applied to estimate the retrieved proteins.

### Chromatin immunoprecipitation (ChIP)

Firstly, MDA-MB-231 and HCC1937 cells which were disposed by 200 ng/ml of EGF or not were collected for the ChIP assay and the number of the cells was 3 × 10^7^. 125 mM glycine was used to neutralize the cells for 5 min after 1% formaldehyde had cross-linked the cells at 37 °C for 10 min. Before the cells were put into 0.3 ml of lysis buffer for resuspension and sonication into 200–1000-bp fragments, they were washed cleanly by ice-cold PBS for two times and taken out for putting into 1 ml of ice-cold PBS. When the centrifugation was completed, IP dilution buffer was use to deliquate the supernatants which were gathered before. And then, they were performed with protein A-Sepharose beads for immune clearing at 4 °C. This process took 2 h. In the process of immunoprecipitation, anti-FOXC1 and control IgG (Cat No: 2729S, CST, Danvers, MA, USA) was taken for experiment. After the process was accomplished, they were added the 45 μl protein A-Sepharose for cultivation at least 1 h. qRT-PCR was conducted to analyze DNA in the precipitates after being washed and purified.

### Luciferase reporter assays

For the activity experiment of promoter, FuGENE 6 (Roche) was used to transfect 500 ng pGL3.0-basic or pGL3.0- LINC01123 vector (Promega, Madison, WI, USA) plus 5 ng of the Renilla luciferase plasmid as control to the cells. Then the cells were incubated with the inhibitors for 24 h after they were transfected for 48 h, so as to estimate the promoter activity of cells which were treated by inhibitors. With regard to the reporter assay of miRNA Luciferase, a 24-well plate was used to seed 293 cells for 24 h until the transfection was up to 50% confluence. The use of Lipofectamine RNAiMAX (Invitrogen) was to transfect with 30 nM of miR-663a mimics or control mimics (Sigma). After the post-transfection was finished at the deadline of 24 h, FuGENE6 transfection reagent (Roche) was used to transfect 0.125 μg of psiCheck- LINC01123 WT or psiCheck- LINC01123 MUT reporter vector. 48 h later, the reporter vector transfection was completed. Finally, under the help of Fluoroskan Ascent FL fluorometer (Thermo Fisher Scientific, Rockford, IL, USA), the activities were processed with the dual luciferase reporter assay system (Promega).

### Statistical analysis

SPSS (SPSS Inc., Chicago, IL, USA) and SAS software were used to take the statistical analysis. All of the results collected from three separately experiments were displayed as mean ± standard deviation (SD). The significance of differences was estimated by the utilization of the Student’s t-test or one-way ANOVA. In addition, P < 0.05 stated clearly the great significance.

## Results

### MiR-663a exerted anti-growth property in TNBC

**Fig. 1:**
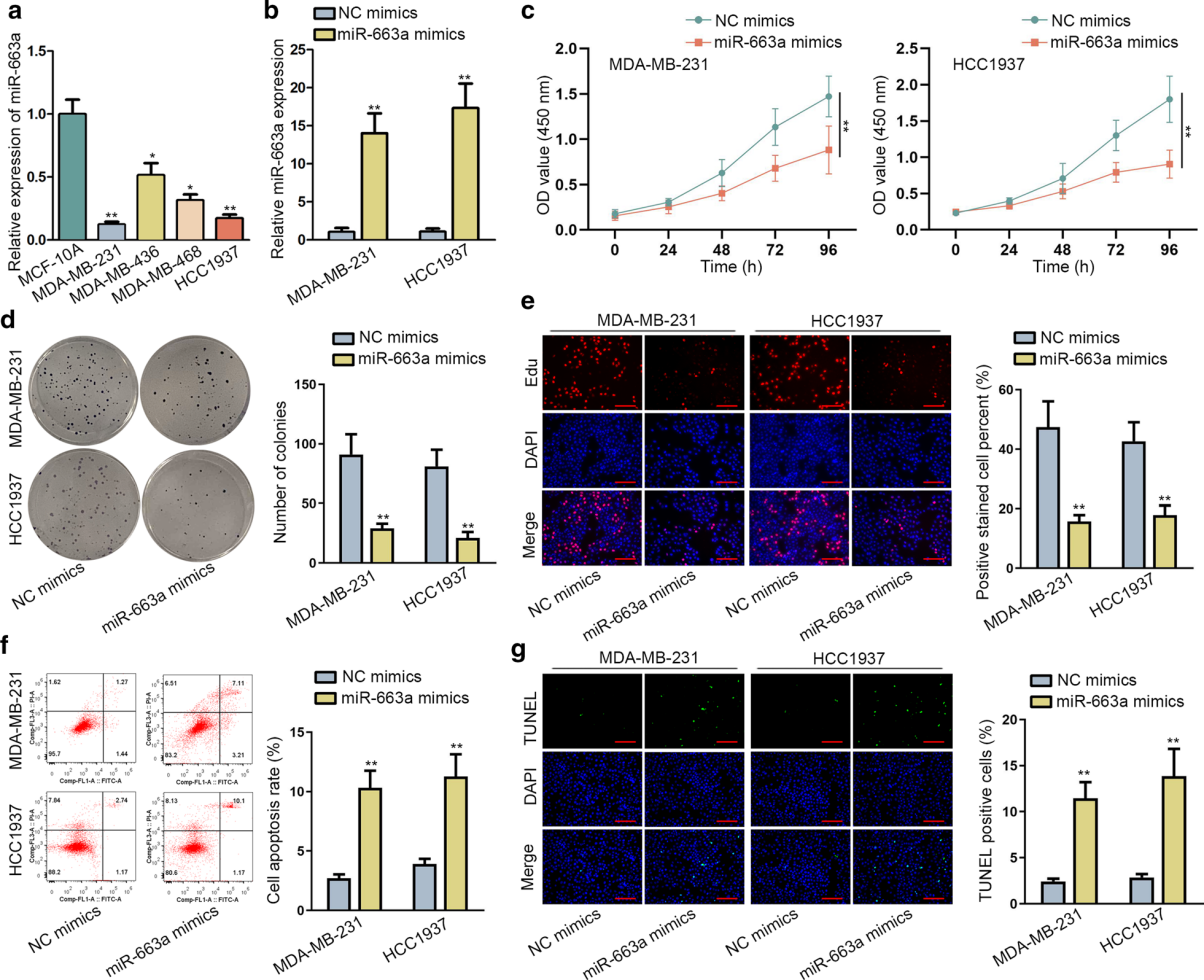
MiR-663a exerted anti-growth property in TNBC. **a** qRT-PCR assay of miR-663a expression pattern in TNBC cell lines and MCF-10A. **b** Overexpression efficiency of miR-663a was validated via qRT-PCR. c–e The effects of miR-663a on TNBC cell growth were evaluated by performing CCK-8, colony formation and EdU assays (scale bar = 100 μm). **f**–**g** Flow cytometry and TUNEL experiments (scale bar = 100 μm) detected the apoptosis rate in miR-663a-overexpressed TNBC cells. Error bar meant the SD of data from three independent experiments. *P < 0.05, **P < 0.01

MiR-663a was characterized as a tumor-implicated miRNA including breast malignancy [22–24]. Thus, we probed its involvement in TNBC. To functionally annotate miR-663a in TNBC cellular growth, we first monitored its expression in TNBC. Experimental from qRT-PCR suggested that miR-663a was reduced in TNBC cell lines (HCC1937, MDA-MB-468, MDA-MB-436 and MDA-MB-231) compared to MCF-10A (Fig. 1a). Considering that miR-663a was suppressed significantly in HCC1937 and MDA-MB-231, we then performed gain-of-function experiments after enforcing miR-663a expression in these two cells (Fig. 1b). Functionally, enhanced miR-663a expression led to attenuated cell viability in two TNBC cells (Fig. 1c). Likewise, overexpression of miR-663a impaired the colony formation and proliferation abilities of HCC1937 and MDA-MB-231 as suggested via colony formation and EdU experiments (Fig. 1d, e). Reciprocally, we observed that augmentation of miR-663a led to the increase in apoptotic rate of HCC1937 and MDA-MB-231 (Fig. 1f, g). All provided evidence that miR-663a was anti-growth in TNBC cellular behaviors.

### LINC01123 regulated miR-663a in TNBC

**Fig. 2:**
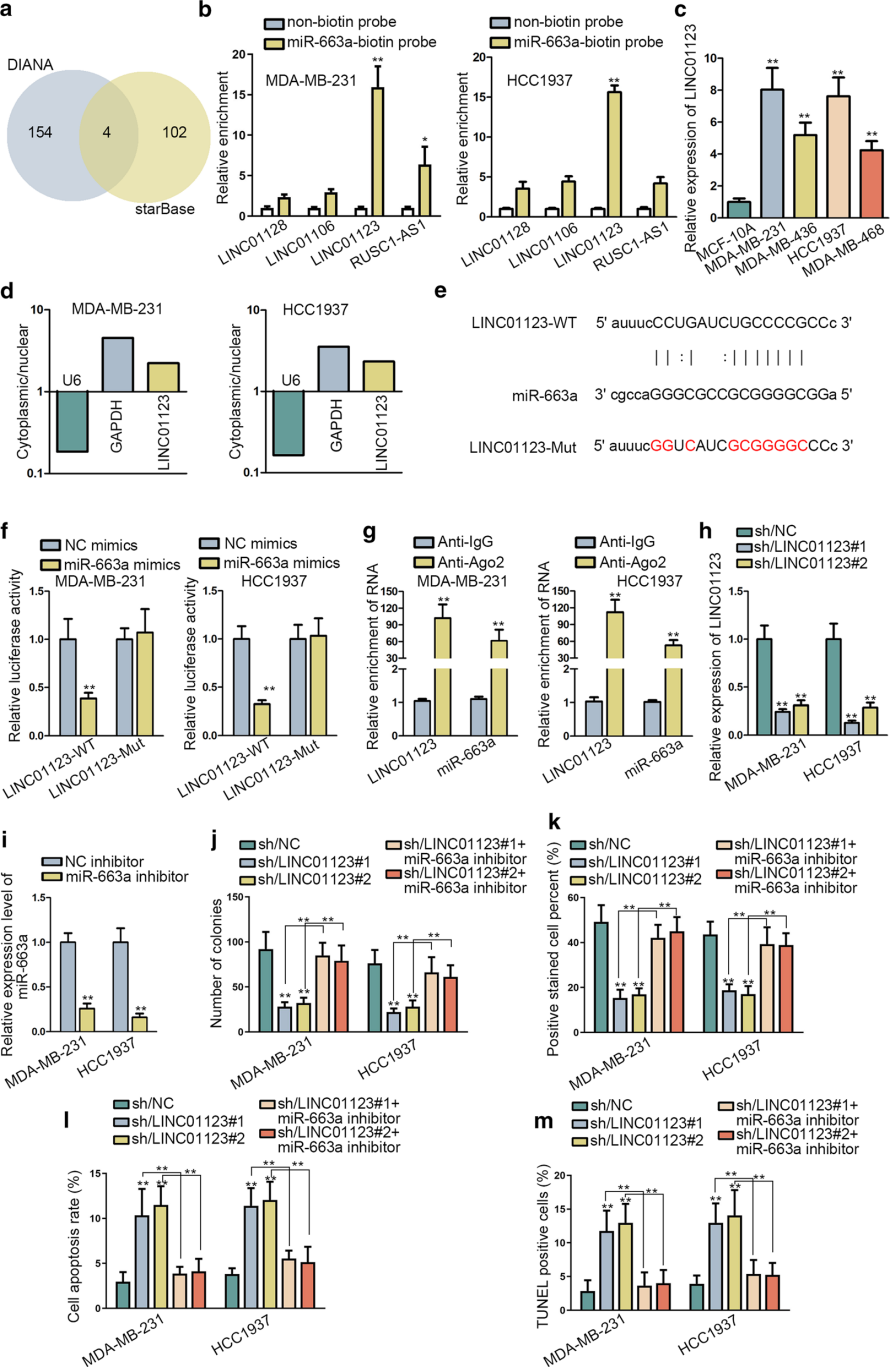
LINC01123 regulated miR-663a in TNBC cell growth. **a** StarBase and DIANA predicted four candidates as upstream lncRNAs of miR-663a. **b** RNA pull-down assay detected the enrichment of four lncRNAs in miR-663a-biotin probe-precipitated group. **c** qRT-PCR analysis of LINC01123 expression in TNBC cell lines and MCF-10A cells. **d** Following the cellular fractionation in TNBC cells, LINC01123 expression from cytoplasm or nucleus was monitored in qRT-PCR. **e** The interaction sequences between LINC01123 and miR-663a predicted by starBase. **f** Luciferase reporter assay in TNBC cells co-transfected with LINC01123 reporters (LINC01123-WT, LINC01123-MUT) and miR-663a mimics or NC mimics. **g** RIP assay showed the precipitation of LINC01123 and miR-663a in Anti-IgG or Anti-Ago2 group. **h**, **i** Knockdown efficacy of LINC01123 and miR-663a in TNBC cells determined by qRT-PCR. **j**, **k** The impacts of LINC01123/miR-663a axis on TNBC cell proliferation were probed by colony formation and EdU assays. l, m Flow cytometry analysis and TUNEL assay reflected the influence of LINC01123/miR-663a pathway on TNBC cell apoptosis. Error bar meant the SD of data from three independent experiments. **P < 0.01

LncRNAs are certified to sponge miRNAs and reduce their ability during tumor progression. Therefore, upstream lncRNAs of miR-663a was searched by means of DIANA (http://carolina.imis.athena-innovation.gr/diana_tools/web/index.php?r=site%2Ftools) and starBase (http://starbase.sysu.edu.cn). Potentially, four lncRNAs were highlighted (Fig. 2a). Furthermore, LINC01123 presented the highest enrichment among the 4 candidates in the compounds pulled down by miR-663a in both MDA-MB-231 and HCC1937 cells, strongly hinting potential interplay between miR-663a and LINC01123 in TNBC (Fig. 2b). Subsequently, qRT-PCR also unveiled the specific increase of LINC01123, but not other three lncRNAs, in TNBC cell lines compared with normal MCF-10A cells (Fig. 2c, Additional file 1: Figure S1A). Besides, we also found that LINC01123 was not dysregulated in hormone dependent breast cancer cell lines (MCF7 and T47D) or HER2-positive breast cancer cell lines (MDA-MB-453 and SKBR3) relative to MCF-10A cells (Additional file 1: Figure S1B). This phenomenon highlighted the specific impact of LINC01123 in TNBC development. Interestingly, we unveiled that LINC01123 was more likely to exist in cytoplasm than in nucleus, supporting its possible post-transcriptional activity in TNBC (Fig. 2d). To further corroborate the LINC01123-miR-663a interaction, luciferase reporter assay was conducted via utilizing luciferase reporters established based on the sequences of LINC01123 recognized by miR-663a as well as corresponding mutant LINC01123 sequences (Fig. 2e). As expected, the luciferase signal of LINC01123-WT in TNBC cells was reduced when co-transfected with miR-663a mimics, whereas no significant effect was revealed when the miR-663a binding site was disrupted (Fig. 2f). Further, as addressed by RIP experiments, Both LINC01123 and miR-663a were precipitated by anti-Ago2, while Ago2 is a core component of RNA-induced silencing complexes (RISCs) (Fig. 2g). To validate whether LINC01123 regulated TNBC cellular growth via miR-663a, we conducted a set of assays. Before that, LINC01123 was down-regulated by two specific shRNAs and miR-663a level was declined using miR-663a inhibitor in HCC1937 and MDA-MB-231 cells (Fig. 2h, i). Of note, it was uncovered that miR-663a inhibition could reverse the suppressed proliferation and induced apoptosis of TNBC cells under LINC01123 depletion by either sh-LINC01123#1 or sh-LINC01123#2 (Fig. 2j, m, Additional file 1: Figure S1C). Overall, LINC01123 sponged miR-663a to facilitate malignant growth of TNBC cells.

### LINC01123 positively regulated oncogenic CMIP in TNBC via miR-663a

**Fig. 3:**
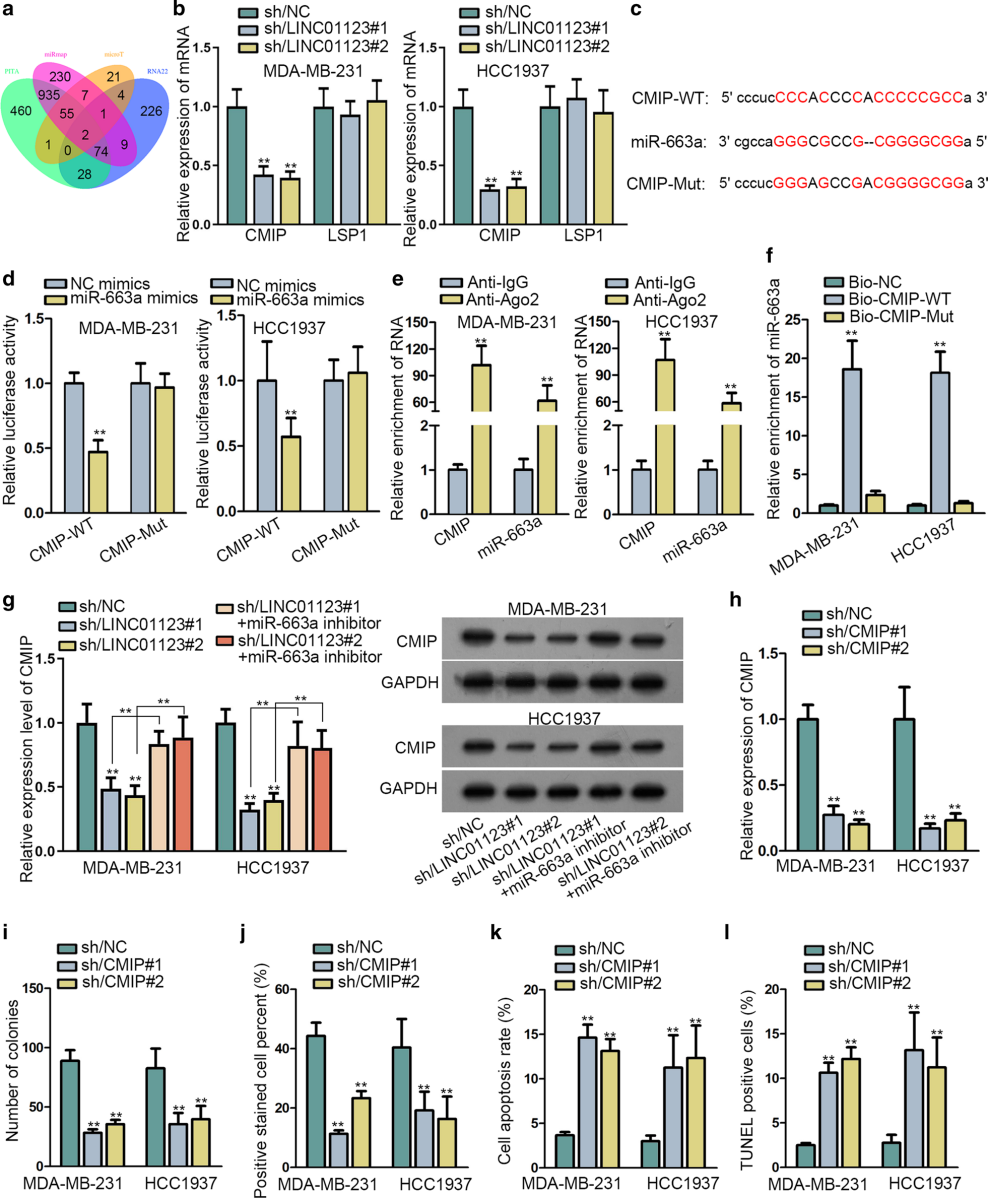
LINC01123 positively regulated oncogenic CMIP in TNBC via miR-663a. **a** Bioinformatics tools disclosed two possible targets of miR-663a. **b** Expression of CMIP and LAP1 in TNBC cells transfected with sh/NC or sh/LINC01123#1/2. **c** The interaction sequences between CMIP and miR-663a was exhibited via starBase prediction. **d** Luciferase reporter assay carried out in TNBC cells co-transfected with indicated reporters (CMIP-WT, CMIP-Mut) and miR-663a mimics or NC mimics. **e** The abundance of CMIP and miR-663a in Anti-IgG or Anti-Ago2 group was assessed via RIP assay plus qRT-PCR. **f** RNA pull down assay indicated the enrichment of miR-663a in Bio-CMIP-WT, Bio-CMIP-Mut or Bio-NC groups. **g** qRT-PCR and western blot examined the mRNA and protein levels of CMIP in MDA-MB-231 and HCC1937 cells under diverse transfections. **h** qRT-PCR revealed the expression of CMIP expression in TNBC cells transfected with sh/NC, sh/CMIP#1 or sh/CMIP#2. **i**, **j** Colony formation and EdU assays conducted in CMIP-depleted cells. **k**, **l** Flow cytometry analysis and TUNEL asaay in CMIP-depleted cells. Error bar meant the SD of data from three independent experiments. **P < 0.01

LncRNAs could indirectly regulate functional genes through sequestering miRNAs from target mRNAs. Therefore, we intended to unravel the miR-663a-targeted mRNAs through various prediction tools. Two candidate mRNAs were introduced via four programs in starBase (Fig. 3a). More importantly, silencing LINC01123 triggered the decline of CMIP (c-Maf inducing protein) not LSP1 in both MDA-MB-231 and HCC1937 cells (Fig. 3b). Also, we discovered the enhancement trend of CMIP in TNBC cells relative to MCF-10A cells (Additional file 1: Figure S1D). The recognition of miR-663a to the sequences of CMIP 3′UTR (3′-untranslated region) was shown in Fig. 3c. Luciferase reporter assay elucidated that co-transfection of miR-663a mimics resulted in the reduction on the luciferase signal of CMIP-WT, while no alteration of luciferase signal of CMIP-Mut was found in TNBC cells (Fig. 3d). Through conducting RIP assay, CMIP and miR-663a were proved to be co-existed in RISCs (Fig. 3e). RNA pull-down further confirmed the direct interaction between miR-663a and CMIP in TNBC cells (Fig. 3f). More interestingly, it was elucidated that augmented LINC01123 could offset the restraining influence of miR-663a mimics on the luciferase activity of CMIP-WT, while the activity of CMIP-Mut still unaffected all the time (Additional file 1: Figure S1E). Besides, LINC01123 upregulation resulted in enhanced enrichment of LINC01123 but reduced harvest of CMIP in Ago2-assembled RISCs in two TNBC cells (Additional file 1: Figure S1F). These two findings further testified the competition between LINC01123 and CMIP for miR-663a binding in TNBC. Later, we uncovered the reduced the mRNA and protein expression of CMIP in sh/LINC01123#1/2-transfected TNBC cells, while co-transfection of miR-663a inhibitor attenuated LINC01123 inhibition-mediated suppression on CMIP (Fig. 3g). Besides, we also probed into the functional characteristics of CMIP in TNBC. As presented in Fig. 3h, shRNAs targeting CMIP (sh/CMIP#1 and sh/CMIP#2) distinctly suppressed CMIP in both MDA-MB-231 and HCC1937 cells. Moreover, it manifested that downregulation of CMIP blocked TNBC cell proliferation but augmented apoptosis (Fig. 3i, l, Additional file 1: Figure S1G). Collectively, carcinogenic CMIP was post-transcriptionally promoted by LINC01123 via sponging miR-663a.

### Post-transcriptional upregulation of CMIP was required in LINC01123-regulated TNBC growth

**Fig. 4:**
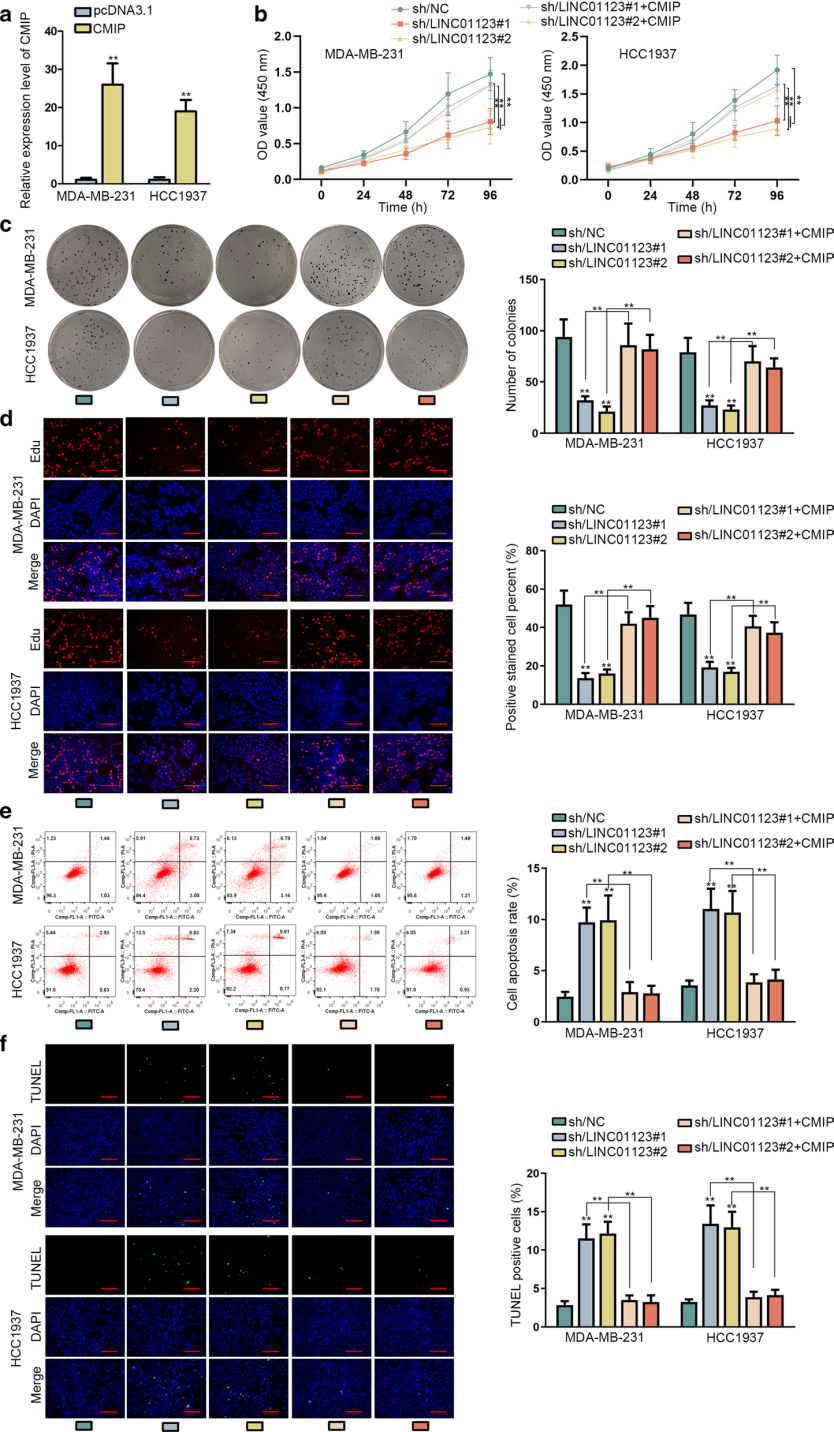
Post-transcriptional upregulation of CMIP was required in LINC01123-regulated TNBC growth. **a** The expression of CMIP expression in TNBC cells transfected with pcDNA3.1 or pcDNA3.1/CMIP. **b**–**d** TNBC cells transfected with sh/NC, sh/LINC01123#1/2, or sh/LINC01123#1/2 + CMIP were analyzed via CCK-8, colony formation and EdU assays (scale bar = 100 μm). **e**, **f** The apoptosis rates of above TNBC cells were measured via flow cytometry analysis and TUNEL assay (scale bar = 100 μm). Error bar meant the SD of data from three independent experiments. **P < 0.01

In order to address our speculation that CMIP was required in LINC01123-regulated TNBC growth, rescue experiments were performed. Prior to that, we ectopically enforced CMIP expression in TNBC cells (Fig. 4a). We dissected that sh/LINC01123#1/2-induced suppression on TNBC growth was overcame by CMIP overexpression (Fig. 4b–d). In the meantime, the increasing trend of apoptosis in LINC01123-silenced TNBC cells could be alleviated via CMIP overexpression (Fig. 4e–f). Taken together, CMIP was indispensable in LINC01123-triggered TNBC cell growth.

### LINC01123 was transcriptionally amplified via FOXC1

**Fig. 5:**
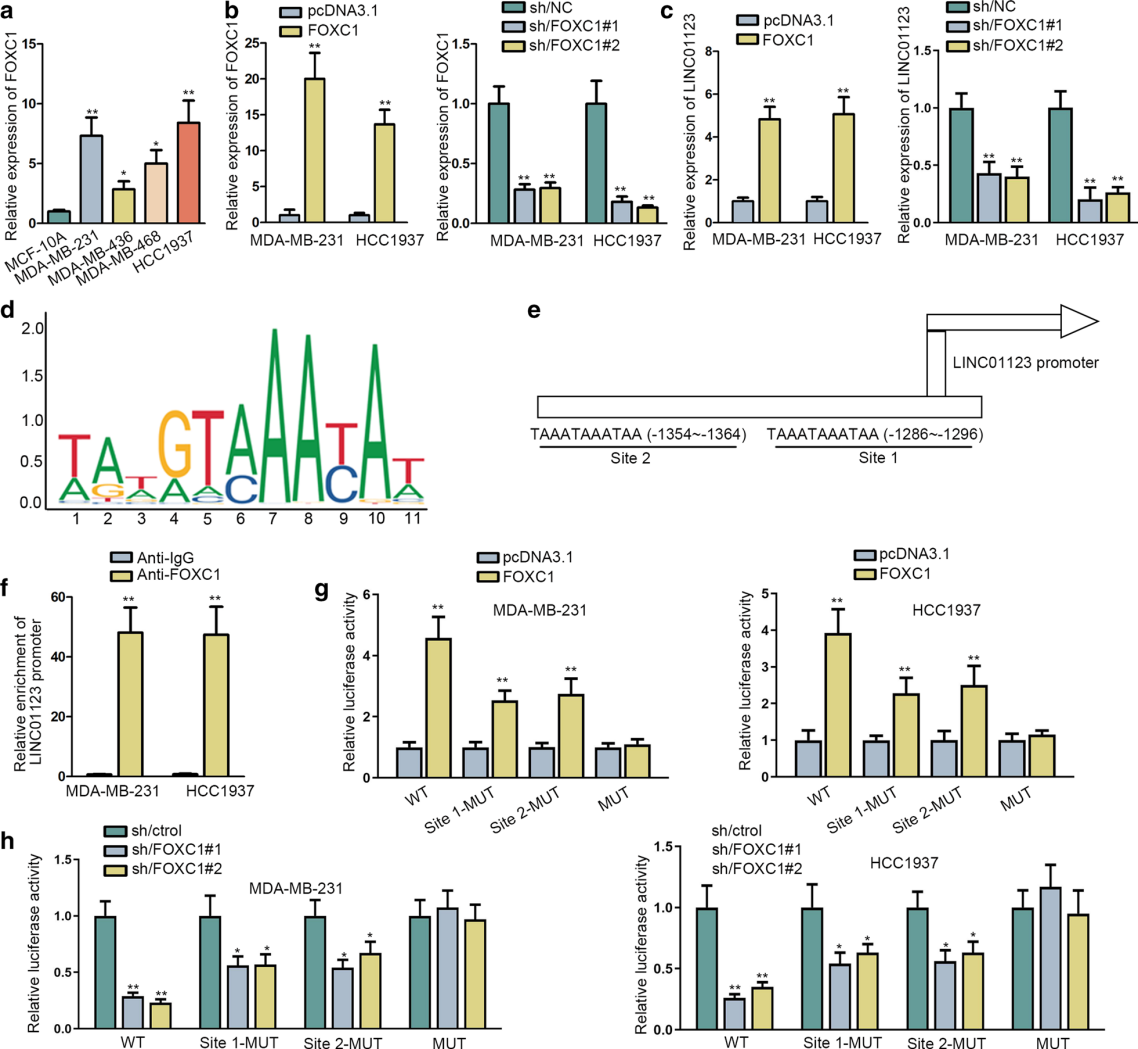
LINC01123 was transcriptionally promoted via FOXC1. **a** FOXC1 expression pattern in TNBC cell lines and MCF-10A was reflected by qRT-PCR. **b** Overexpression or suppression of FOXC1 was effectively conducted, as evidenced by qRT-PCR. **c** The influence of FOXC1 on LINC01123 expression was determined via qRT-PCR. **d**, **e** JASPAR provided FOXC1 motif and also predicted the binding region of FOXC1 in LINC01123 promoter. The sequences of LINC01123 promoter were obtained from UCSC. **f** ChIP assay followed by qRT-PCR monitored the abundance of LINC01123 promoter in immunoprecipitates induced by antibodies against FOXC1 or IgG. **g**, **h** Luciferase reporter assay showed the impact of FOXC1 overexpression or depletion on LINC01123 transcription in TNBC cells. Error bar meant the SD of data from three independent experiments. *P < 0.05, **P < 0.01

To dissect the probable mechanism for overexpressed LINC01123 in TNBC, we spotted on TNBC-related transcriptional factor FOXC1 (forkhead box C1) [25]. Firstly, we confirmed the elevation of FOXC1 in TNBC cell lines (Fig. 5a). Then, we wondered whether FOXC1 was responsible for LINC01123 in TNBC and results presented that FOXC1 elevation led to an increase of LINC01123 level while FOXC1 repression obviously reduced LINC01123 level (Fig. 5b, c). From the perspective bioinformatics revelation, JASPAR (http://jaspar.genereg.net/) demonstrated the FOXC1 motif and its potent binding region in LINC01123 promoter (site 1, site 2; score threshold 95%) (Fig. 5d, e). The sequences of LINC01123 promoter (with 2000 bp in length) upstream the transcriptional start site (TSS) were obtained from UCSC (http://genome.ucsc.edu/). Then, ChIP assay depicted the robust occupancy of FOXC1 in LINC01123 promoter (Fig. 5f). Afterwards, luciferase reporter assays showed that FOXC1 overexpression increased the luciferase activity of vectors with whole LINC01123 promoter. Compared to this, mutations at site 1 or site 2 led to the slightly weakened increase of the luciferase activity of LINC01123 promoter following FOXC1 overexpression. Moreover, when site 1 and site 2 were both mutated, the luciferase activity of LINC01123 promoter remained unaffected in FOXC1-upregulated TNBC cells (Fig. 5g). These results indicated that FOXC1 modulated LINC01123 transcription in TNBC through binding to both site 1 and site 2, and such deduction was further validated by the phenomenon observed in TNBC cells with ablation of FOXC1 (Fig. 5h). All observations suggested that FOXC1 as the transcriptional inducer of LINC01123 in TNBC.

### Enforced LINC01123 reversed the growth of FOXC1-depleted TNBC cells

**Fig. 6:**
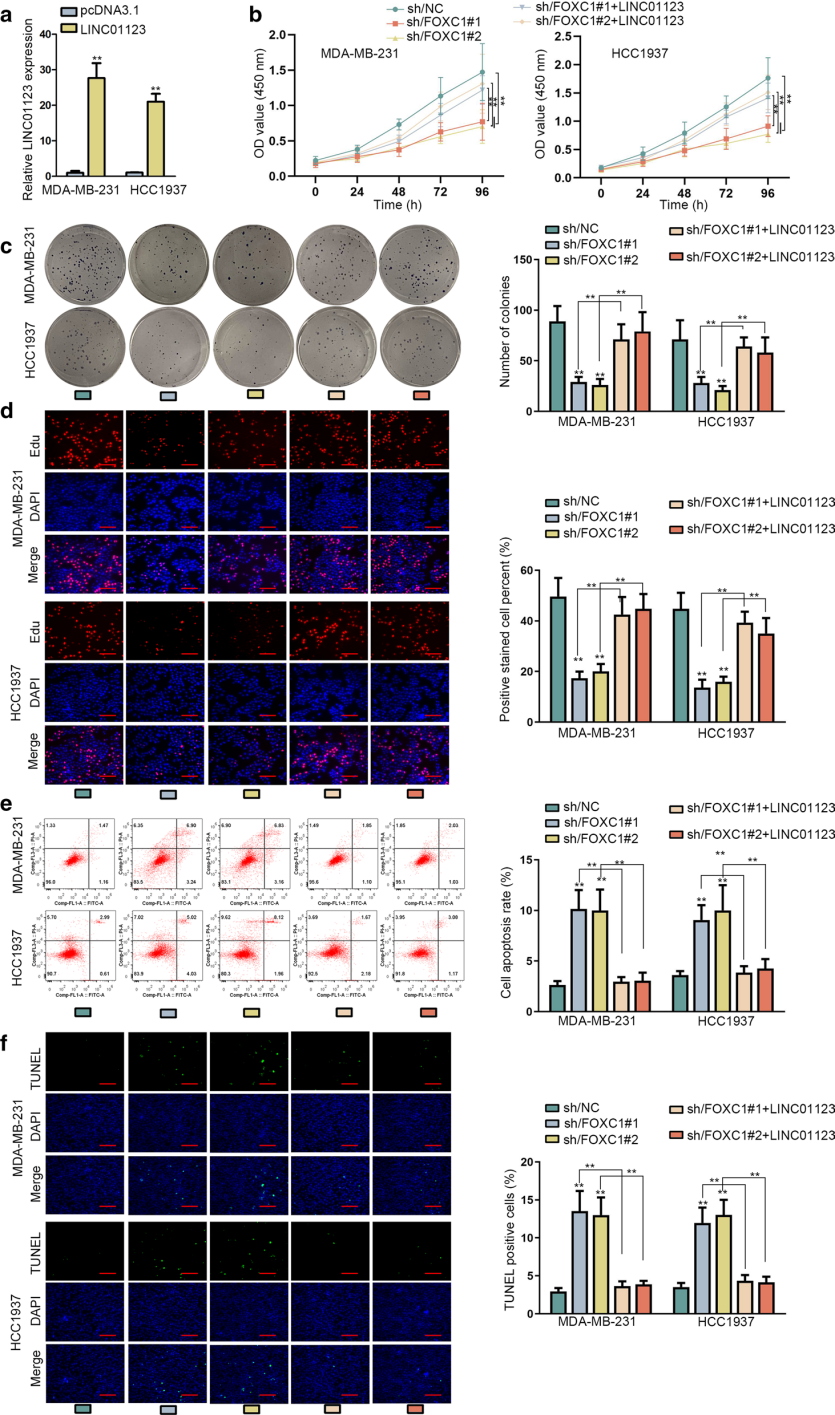
Enforced LINC01123 reversed the hindered growth of FOXC1-depleted TNBC cells. **a** The expression of LINC01123 in TNBC cells transfected with pcDNA3.1 or pcDNA3.1/LINC01123 was analyzed via qRT-PCR. **b**–**d** TNBC cells transfected with sh/NC, sh/FOXC1#1/2, or sh/FOXC1#1/2 + LINC01123; CCK-8, colony formation and EdU assays (scale bar = 100 μm) were implemented to investigate changes on proliferation in above TNBC cells. **e**, **f** Flow cytometry analysis and TUNEL assay (scale bar = 100 μm) probed the apoptosis in indicated TNBC cells. Error bar meant the SD of data from three independent experiments. **P < 0.01

Based on previous validation that FOXC1 directly induced LINC01123 expression, we further consolidated the function of FOXC1/LINC01123 pathway in TNBC. First of all, we overexpressed LINC01123 in TNBC cells (Fig. 6a). Afterwards, we unveiled that silencing FOXC1 greatly blunted TNBC cell viability and proliferation while augmentation of LINC01123 overcame above suppressive trend (Fig. 6b–d). Furthermore, the pro-apoptosis role of FOXC1 downregulation could be abolished via overexpressing LINC01123- (Fig. 6e–f). Altogether, FOXC1-activated LINC01123 was involved in TNBC cellular growth.

## Discussion

Aggressiveness of TNBC makes it as a malignancy with higher recurrence together with mortality rate [2]. Despite the clinical progress in hormone therapy for luminal subtype breast cancer patients and trastuzumab therapy for HER2 subtype treatment, specific feasible treatments for TNBC patients are limited [26]. At present, a comprehensive portrait of molecular mechanism is in dire need in TNBC oncogenesis. Accumulating works proposed that miRNAs with specific expression is tumor-related. In early works, miR-663a acts as a tumor suppressor in hepatocellular carcinoma [22], non-small cell lung cancer [27] and pancreatic cancer [23]. Meanwhile, it is oncogenic in renal cell carcinoma, which denotes malignant cellular processes and worse prognosis [28]. In breast cancer, miR-663a is downregulated based on prior research [24]. Herein, we first portrayed that miR-663a was evidently inhibited in TNBC cell lines. Moreover, overexpression of miR-663a effectively restrained the malignant growth of TNBC cells and conversely resulted in facilitated apoptosis, which provided further evidence for miR-663a participation in TNBC.

Motivated by the lncRNAs-mediated the decline in miRNA availability, we speculated that miR-663a was also under the regulation of a certain lncRNA in TNBC. Through bioinformatics assays, we identified LINC01123 as a possible upstream factor of miR-663a. As annotated before, highly expressed LINC01123 overtly reduces the survival of head neck squamous cell carcinoma [29]; it also aggravates the uncontrolled proliferation of non-small cell lung cancer cells [30]. In our work, mechanical data supported that LINC01123 could interact with miR-663a. Besides, downregulation of miR-663a reverse the suppressive proliferation of TNBC cells due to the suppression of LINC01123. Overall, the ceRNA role of LINC01123 was first introduced in TNBC, accompanied with its oncogenic nature.

CMIP is crucial in negatively regulating T-cell signaling pathway [31]. The participation of CMIP is observed in multiple cellular processes, such as chondrocyte terminal differentiation and cell growth in embryonic lens. Also, CMIP has been reported to have considerable contributions in tumors. For instance, it has been proved to be pro-metastasis and pro-proliferation in gastric cancer [32] and glioma [33]. Furthermore, overexpressed CMIP inversely correlated with breast cancer patients’ survival [34]. In this work, we demonstrated that CMIP was targeted by miR-663a in TNBC. The positive regulation of LINC01123 on CMIP was also confirmed. Functionally, CMIP suppression led to TNBC proliferation retardation and apoptosis enhancement. All findings in this work suggested that LINC01123 exerted its function in TNBC through a CMIP-dependent way via sponging miR-663a. More importantly, we also gave a more detailed description about the oncogenic property of CMIP in TNBC.

For the exploration of LINC01123 upregulation in TNBC, we unveiled that forkhead box C1 (FOXC1) directly amplified the expression of LINC01123 through transcriptional regulation. On a global scale, FOXC1 is identified as a transcriptional amplifier of diverse genes, such as WNT5A [25] and PTGER3 [35]. Prior studies summarized that FOXC1 is a tumor facilitator in breast cancer [36], cervical carcinoma [37], esophageal cancer [38] and melanoma [39]. Of interest, FOXC1 has also been uncovered to be overexpressed in TNBC [25]. Herein, the promoting effect of FOXC1 on LINC01123 opens up a new page of its activity in TNBC progression.

## Conclusion

In summary, we determined that FOXC1-induced LINC01123 upregulated oncogenic CMIP via sponging miR-663a to contribute to TNBC, highlighting its significance in enriching the molecular basis in TNBC. Furthermore, future works will pour great attention to the clinical value of LINC01123 in TNBC.

## Supplementary information

**Additional file 1: Figure** **S1.** (A) The expression of LINC01128, LINC01106 and RUSC1-AS1 in TNBC cells in comparison with MCF-10A cells was estimated by qRT-PCR. (B) The expression of LINC01123 in hormone dependent breast cancer cell lines (MCF7 and T47D) and HER2-positive breast cancer cell lines (MDA-MB-453 and SKBR3) normalized to MCF-10A cells was assessed by qRT-PCR. (C) CCK-8 assay tested the viability of TNBC cells with transfection of sh/NC, sh/LINC01123#1/2, or sh/LINC01123#1/2 + miR-663a inhibitor. (D) qRT-PCR revealed the expression of CMIP in TNBC cells and MCF-10A cells. (E-F) Luciferase reporter and RIP assays disclosed the competition between LINC01123 and CMIP for miR-663a interaction in RISCs. (G) The viability of CMIP-inhibited TNBC cells was assayed via CCK-8. Error bar meant the SD of data from three independent experiments. **P < 0.01.

## Data Availability

Research data and material could be accessed to under reasonable requirements.

## Abbreviations

TNBC: Triple negative breast cancer
MiRNAs: MicroRNAs
HER-2: Human epidermal growth factor receptor-2
HR: Hormone receptor
lncRNAs: Long non-coding RNAs
mRNA: Messenger RNA
miR-663a: microRNA-663a
qRT-PCR: Quantitative real-time polymerase chain reaction
TUNEL: Terminal deoxynucleotidyl transferase-mediated dUTP nick end labeling
EdU: 5-Ethynyl-2′-deoxyuridine
RIP: RNA-immunoprecipitation
ChIP: Chromatin immunoprecipitation
LINC01123: Long intergenic non-protein coding RNA 1123
FOXC1: Forkhead box C1
CMIP: c-Maf inducing protein
