# Supplementary figures and images for: FOXC1-induced LINC01123 acts as a mediator in triple negative breast cancer
Source: Cancer Cell Int. 2020 May 29;20:199. doi: 10.1186/s12935-020-01258-z (PMC7257197; doi:10.1186/s12935-020-01258-z)

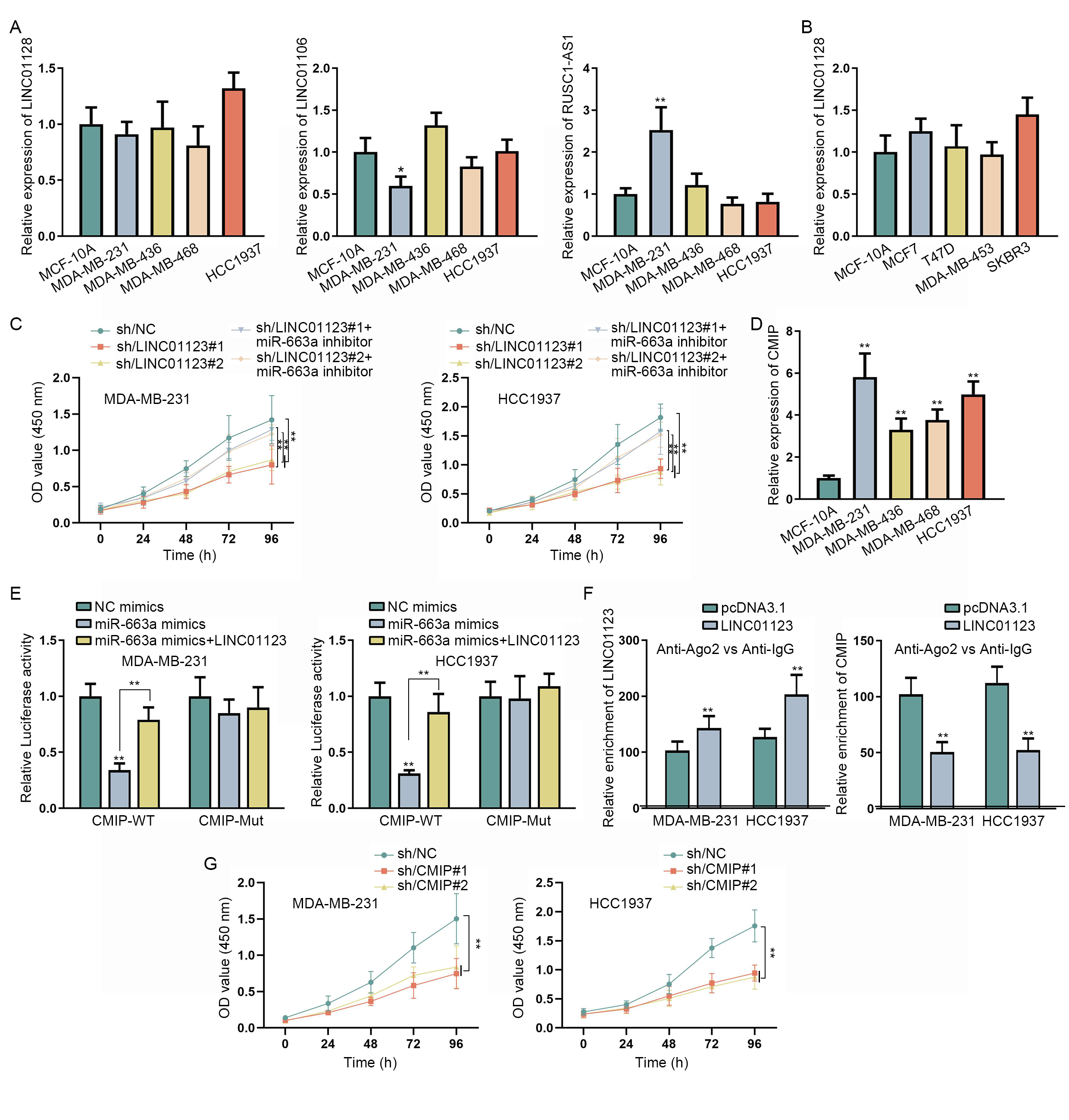

Supplement: Supplementary file 1 — Additional file 1: Figure S1. (A) The expression of LINC01128, LINC01106 and RUSC1-AS1 in TNBC cells in comparison with MCF-10A cells was estimated by qRT-PCR. (B) The expression of LINC01123 in hormone dependent breast cancer cell lines (MCF7 and T47D) and HER2-positive breast cancer cell lines (MDA-MB-453 and SKBR3) normalized to MCF-10A cells was assessed by qRT-PCR. (C) CCK-8 assay tested the viability of TNBC cells with transfection of sh/NC, sh/LINC01123#1/2, or sh/LINC01123#1/2 + miR-663a inhibitor. (D) qRT-PCR revealed the expression of CMIP in TNBC cells and MCF-10A cells. (E-F) Luciferase reporter and RIP assays disclosed the competition between LINC01123 and CMIP for miR-663a interaction in RISCs. (G) The viability of CMIP-inhibited TNBC cells was assayed via CCK-8. Error bar meant the SD of data from three independent experiments. **P < 0.01. [file 12935_2020_1258_MOESM1_ESM.tif]
